# Assessing the effect of climatic factors on the childhood diarrhea burden in four districts of Nepal

**DOI:** 10.1016/j.joclim.2026.100746

**Published:** 2026-09-26

**Authors:** Bimala Dhimal, Rijan Bhakta Kayastha, Biraj Man Karmacharya

**Affiliations:** a Department of Environmental Science and Engineering, Kathmandu University, School of Science, Dhulikhel, Nepal; b Department of Public Health, Kathmandu University, School of Medical Sciences, Dhulikhel, Nepal

**Keywords:** Climate change, Diarrheal disease districts, Province, Nepal

## Abstract

**Introduction:**

This study examines the impact of climatic factors on the burden of diarrheal disease among children under the age of five living in the Dhankuta, Morang, Surkhet, and Jajarkot districts of Nepal.

**Method:**

A time series study analyzed the monthly case counts of diarrheal disease among children under five years from 2002 to 2022. A generalized linear model with a quasi-Poisson distribution was applied to examine the relationship between climatic variables and diarrheal disease incidence.

**Results:**

In the Jajarkot district, the monsoon season was associated with a 26% increase in diarrheal disease incidence compared with non-monsoon seasons. In the Surkhet district, each 1 °C increase in maximum temperature was associated with a 4.7% increase in diarrheal cases, while each 1 °C increase in minimum temperature was associated with a 4.9% increase in diarrheal incidence. Similarly, in the Morang district, each 1 °C increase in maximum temperature was associated with a 9.6% increase in diarrheal cases, whereas each 1 °C increase in minimum temperature was associated with a 7% increase in diarrheal incidence. In the Dhankuta district, each 1 °C increase in minimum temperature was associated with a 10.3% increase in diarrheal incidence. Additionally, each 1 mm increase in rainfall was associated with a 0.9% increase in diarrheal disease incidence.

**Conclusion:**

Our research identifies and quantifies the relationship between climate factors and the positive association of diarrheal disease. The findings suggest that ongoing climate change could contribute to increased infectious diseases in Nepal and the region. These findings provide evidence to inform public health prevention programs aimed at reducing diarrheal disease-related morbidity and mortality in South Asia.

## 1 Introduction

Diarrheal disease is a leading cause of death in children under five years of age, with nearly 1.7 billion cases reported each year globally by the World Health Organization (WHO) [1], underscoring its major public health impact. It also remains the second leading cause of mortality in the under-five age group in low- and middle-income countries (LMICs), accounting for 1.31 million diarrheal-related deaths worldwide in 2015 [2,3]. Although, the global mortality rate from diarrheal diseases has declined over the past few decades, this trend has not been uniform across all regions [4,5]. In Nepal, diarrheal disease morbidity among children under five remains high [6]. According to the 2022 Nepal Demographic and Health Survey (NDHS) report, the prevalence of diarrheal disease among children under five is 10% [7]. Globally, children under five are the most affected by diarrheal disease, experiencing the highest levels of morbidity and mortality. Children under five years of age are particularly vulnerable due to undeveloped immunity and higher susceptibility to dehydration, making them a key target for public health prevention and treatment programs. Although childhood diarrheal diseases are driven by many factors, including unsafe water, poor sanitation, and limited healthcare, understanding their relationship with climate is equally important. Climate change-related extreme rainfall and temperature increases are likely to increase the occurrence of diarrheal diseases (bacterial and protozoan) via different mechanisms. An increase in temperature favors the replication and survival of pathogens in the intermediate substrate (soil, food, and water) or their zoonotic hosts and may cause overexpression of virulence genes. An increase in precipitation flushes pathogens into the waterways during rainfall events or concentrates pathogens in groundwater reservoirs or surface water bodies during droughts [[8], [9], [10], [11]]. Additionally, evidence suggests that the climate variables of temperature and rainfall influence the development and spread of diarrheal diseases [[12], [13], [14]]. Therefore, recognizing the relevant climate–health links helps improve prevention strategies and better protect vulnerable children, especially in resource-limited settings.

Nepal’s experience with childhood diarrheal cases is broadly relevant to other LMICs, especially in South Asian countries, highlighting common risk factors and challenges [15,16]. Only a few published studies have specifically examined the relationship between climatic factors and diarrheal disease in specific regions of Nepal [17,18]. In addition, the prevalence of diarrheal cases within two weeks varied geographically across the country, ranging from 3.7% to 9.0% in different provinces [19]. Therefore, we conducted this study to estimate the effects of climate factors on the burden of childhood diarrheal disease and to evaluate the impact of climate variability in four districts of the Koshi and Karnali provinces of Nepal. The primary objective was to provide valuable insights for policymakers by assessing how the risk of diarrheal disease among children under five in these provinces varies under the influence of climate change.

## 2 Materials and methods

### 2.1 Study area

Table 1.

**Table 1:** Geographical and climatic characteristics of the study provinces in Nepal.

**Province**	**Study Districts**	**Location**	**Elevation Range**	**Climate Characteristics**	**Key Geographic Features**	**Notes on Comparability**	**Climate–Diarrheal Disease Relevance**
**Koshi Province**	Morang, Dhankuta	Eastern Nepal	∼60 m to >8,000 m	Humid subtropical in lowlands; temperate to alpine at higher elevations	Includes Terai plains, mid-hills, and Himalayan regions; home to Mt. Everest	Comparable to regions with mixed lowland–mountain environments and monsoon climates	Warm, humid lowlands and heavy monsoon rainfall increase risk of water contamination and diarrheal disease; flooding can compromise water quality, contributing to higher seasonal case counts [8].
**Karnali Province**	Surkhet, Jajarkot	Western Nepal	∼500 m to >7,000 m	Predominantly cold temperate to alpine; semi-arid zones in trans-Himalayan areas	Highly mountainous and sparsely populated; includes high-altitude valleys	Comparable to high-altitude or rural regions with limited infrastructure	Diarrheal disease risk is influenced by seasonal water scarcity, reliance on untreated surface water, infrastructure challenges, and disrupted water access during heavy rains or landslides [20]

### 2.2 Study site selection

The four study districts (Morang, Dhankuta, Surkhet, and Jajarkot) were purposively selected to represent diverse ecological zones and climatic conditions across the Koshi and Karnali Provinces of Nepal. Morang represents the lowland Terai ecology, Dhankuta the mid-hill region, Surkhet the inner Terai/valley setting, and Jajarkot the higher hill region. These districts differ substantially in altitude, temperature, precipitation patterns, access to health services, and population density, enabling assessment of diarrheal responses to climatic variability across heterogeneous settings.

### 2.3 Research design

A cross-sectional retrospective study was designed to assess the association between diarrheal disease in children under the age of five and climatic factors (temperature, rainfall, and relative humidity) in the designated four districts of Nepal between 2002 and 2022.

### 2.4 Data sources

The data used in the study were obtained from different sources. The monthly reported diarrheal cases among children under 5 years of age living within the Morang, Dhankuta, Surkhet, and Jajarkot districts of the Koshi and Karnali Provinces, over a 20-years period (2002–2022), were included as the primary outcome variables. Data on the monthly count of diarrheal disease were obtained from the Health Management Information Section (HMIS), Department of Health Services (DoHS), Ministry of Health and Population, Nepal. Monthly diarrheal case data were obtained from local health facilities across all provinces and compiled through the Health Management Information System (HMIS), where the data are aggregated at both the district and national levels and are reported monthly. Individual-level attributes such as age, income, type of sanitation system, or immune-compromised status were not available and could not be included in the analysis. In addition, the HMIS dataset does not record details of the specific diarrheagenic pathogens by which population members (children under five years of age) have been infected. As such, our analysis included the aggregated monthly diarrheal disease count as the outcome variable in our regression model during the data analysis. The meteorological data used in the study were obtained from the Department of Hydrology and Meteorology (DHM), Nepal. Monthly average climate data on maximum temperature, minimum temperature, rainfall, and relative humidity (RH) of the Morang, Dhankuta, Surkhet and Jajarkot districts were obtained for the study duration (2002–2022). We selected the specific climate factors (maximum temperature and minimum temperature, rainfall, and RH) because they are widely recognized in the literature as key determinants of diarrheal disease risk. These variables influence pathogen survival, transmission, and water quality, and are commonly used in various epidemiological research examining climate–health relationships [8,17].

### 2.5 Statistical analysis

Descriptive statistics were used to summarize the study variables, including measures of mean, median, minimum, maximum, first quartile, and third quartile. Monthly meteorological variables and diarrheal case counts were categorized into three seasons: pre-monsoon (February–May), monsoon (June–September), and post-monsoon (October–January).

To assess the association between climatic factors and diarrheal incidence, a generalized linear model (GLM) with a quasi-Poisson distribution and log link function was fitted separately for each district. The quasi-Poisson model was selected to account for overdispersion in monthly diarrheal case counts, where the variance exceeded the mean. The model included maximum temperature, minimum temperature, rainfall, relative humidity, and season as explanatory variables. Season was included as a categorical predictor in the GLM, enabling estimation of seasonal differences in diarrheal incidence (pre-monsoon, monsoon, and post-monsoon) within the same multivariable regression framework, rather than through separate mean comparison tests.

The expected monthly diarrheal incidence was modeled using a quasi-Poisson generalized linear model with a log link:

log(E[Diarrheal_incidence])=β0+β1(Max_temp)+β2(Min_temp)+β3(Rainfall)+β4(RH)+β5(Season)

Regression coefficients (β) from the fitted models represent the log change in the expected count of diarrheal cases associated with a one-unit increase in the corresponding climatic variable (1 °C for temperature and 1 mm for rainfall), holding other variables constant. For interpretability, coefficients were exponentiated and expressed as percent change in diarrheal incidence, calculated as (exp (β) −1) × 100. The corresponding 95% confidence intervals were derived by applying the same transformation to the lower and upper bounds of the coefficient confidence intervals.

P-values were obtained directly from the quasi-Poisson GLM using Wald tests. All statistical analyses were conducted using R software, and statistical significance was assessed at the 5% level.

### 2.6 Ethical approval

The Ethical Review Board (ERB) of the Nepal Health Research Council (NHRC) approved the protocol of this study (registration no 574/2022).

## 3 Results

### 3.1 Descriptive analysis of diarrheal case counts and climatic variables in the study area

A total of 1,209,499 cases of diarrheal disease were reported among the children under 5 years of age living in the four included districts during the study period from 2002 to 2022. The monthly average diarrheal case count reported during the study period was 2,168 (Morang), 475 (Dhankuta), 1,645 (Surkhet), and 628 (Jajarkot) (Table 2). The overall trend of climatic factors of the study districts from 2002–-2022 shows that Morang district had the highest temperature, with means of 30.5⁰C in maximum and 19.4⁰C in minimum temperature. The highest monthly mean rainfall was also received by Morang (1,000.1 mm), followed by Dhankuta (878.1 mm) and Surkhet (799.3 mm) measured throughout the year (Table 2). However, the Jajarkot district received a high amount of rainfall during the monsoon seasons (Fig. 2). Similarly, the RH of Jajarkot is relatively higher, with a mean of 77.21% throughout the year and fluctuating during different seasons.

**Table 2:** Monthly average distribution of meteorological data and diarrheal cases in children under five years in study districts (2002–2022).

Districts	Statistic	Diarrheal cases	Maximum Temperature (⁰C)	Minimum Temperature (⁰C)	Rainfall (mm)	Relative Humidity (%)
Morang	Lowest	33	18.8	7.7	0	52.7
	1st quartile	487	28.6	14	1.6	72.1
	Median	2,250	32.0	21.3	46.2	79.3
	Mean	2,168	30.5	19.4	147.4	76.7
	3rd quartile	3,678	33.1	25.2	236.8	83.3
	Highest	6,281	36.7	26.8	1,000.1	91.0
Dhankuta	Lowest	45	17.5	5.7	0	50.1
	1st quartile	138.2	23	10.9	6	70.2
	Median	296	26.7	15.9	51.1	76.0
	Mean	475	25.5	15.2	117.4	76
	3rd quartile	622.8	28.1	20.1	181	83.5
	Highest	2,806	31.4	21.8	878.1	89.9
Surkhet	Lowest	26	18.9	3.3	0	36.3
	1st quartile	513.8	25.1	9.3	6.0	66.4
	Median	861.5	30.1	16.5	48.1	77.7
	Mean	1,645.1	29.0	15.5	132.5	73.4
	3rd quartile	2957	32.3	22.4	188.4	82.8
	Highest	6755	38.6	24.8	799.3	92.6
Jajarkot	Lowest	39.3	17.8	0.3	0	39.3
	1st quartile	300.5	23.4	8.3	5.5	66.2
	Median	506.5	28.0	15.4	48.1	80.9
	Mean	628.2	27.3	14.4	139.5	77.2
	3rd quartile	750.5	30.3	20.2	235.7	88
	Highest	3,246	38.8	23.3	755.1	98.1

### 3.2 Trend of meteorological data and diarrheal cases according to season

Seasonal patterns of meteorological variables across the four study districts are presented in Fig. 1. The maximum and minimum temperatures exhibited regional variations, with the Morang district generally experiencing higher temperatures. Rainfall was concentrated in the monsoon season, with some variations in intensity across districts. RH was highest during the monsoon and varied across seasons and districts. Similarly, Fig. 2 illustrates the total diarrheal cases by season (Pre-monsoon, Monsoon, and Post-monsoon) from 2002 to 2022 across the four districts. Diarrheal cases were highest during the monsoon season in all districts. Morang and Surkhet experienced a significantly higher diarrheal disease incidence compared to Dhankuta and Jajarkot. However, after 2015 the diarrheal cases exhibited a declining trend (Fig. 2).

**Fig. 1:**
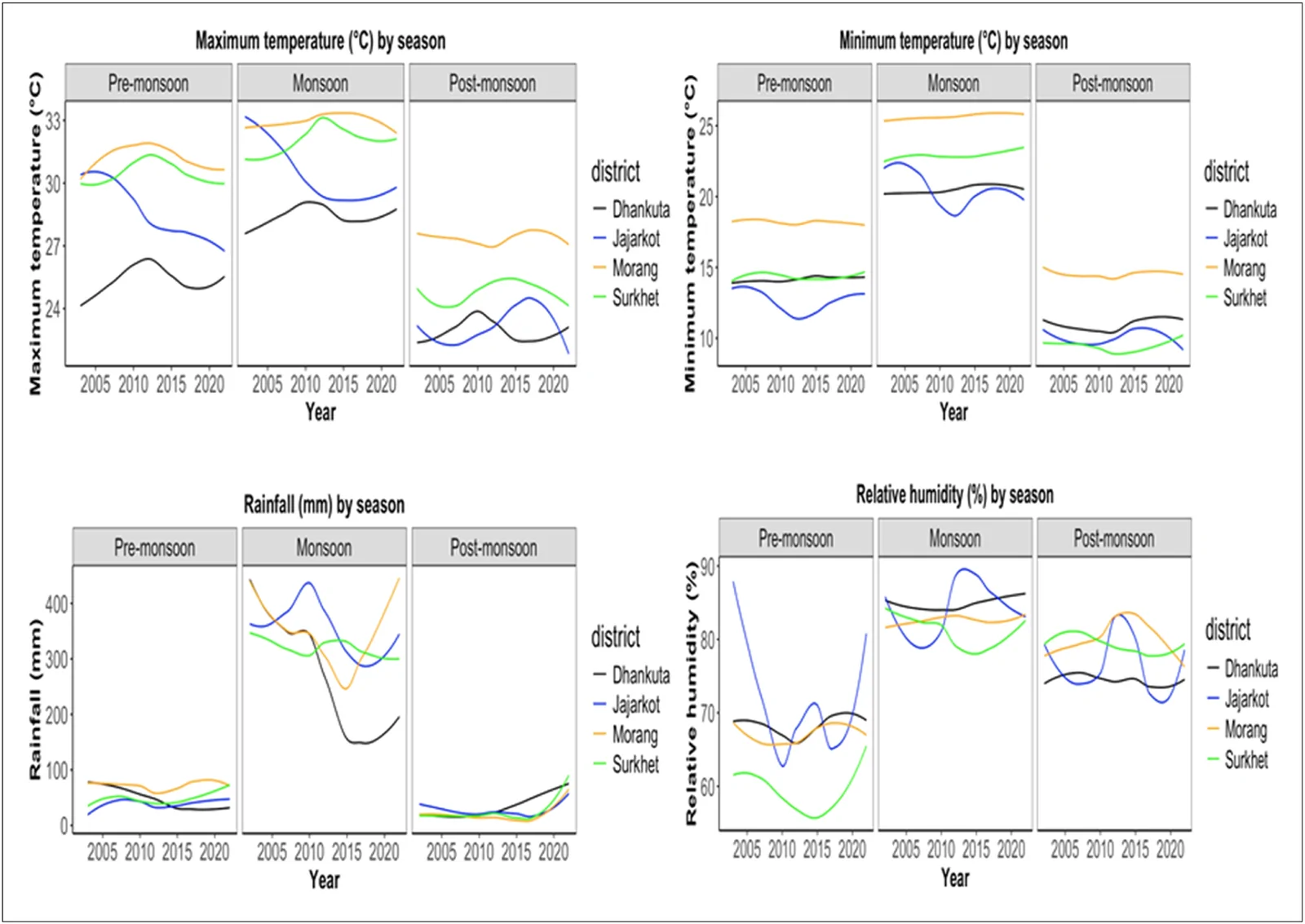
Seasonal trends of meteorological data in the Dhankuta, Jajarkot, Morang, and Surkhet districts (2002–2022).

**Fig. 2:**
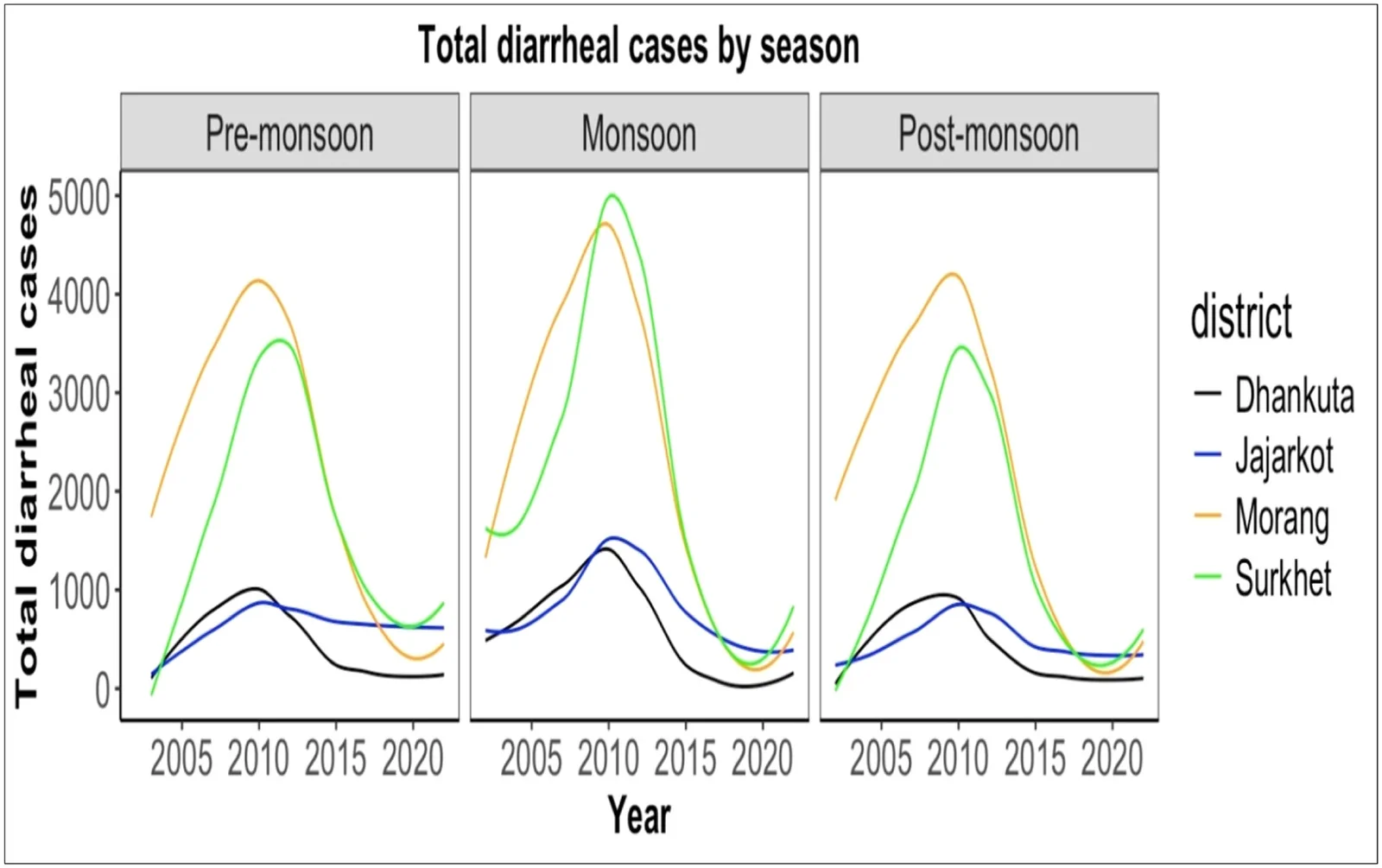
Trends of diarrheal cases in the Dhankuta, Jajarkot, Morang, and Surkhet districts (2002–2022).

### 3.3 Association between climate factors and diarrheal diseases

The study examined how the magnitude of monsoon-related diarrheal disease incidence changed over time and differed across districts. In the quasi-Poisson GLM, monsoon season was significantly associated with a 26% increase in reported diarrheal incidence in the Jajarkot district relative to the non-monsoon reference category, after adjustment for temperature, rainfall, and relative humidity (95% CI: 3.2%–81.9%; *P* < 0.001). No significant associations were observed for maximum temperature, minimum temperature, rainfall, relative humidity, or the post-monsoon and pre-monsoon seasons in the Jajarkot district (*P* > 0.05). The diarrheal case incidence of the Surkhet district is higher in monsoon season by 14.1% compared to the pre- and post-monsoon season. In the Surkhet district, model estimates from the quasi-Poisson GLM showed positive associations between temperature variables and reported diarrheal incidence. Each 1 °C increase in maximum temperature was associated with a 4.7% increase in reported diarrheal incidence (95% CI: 2.6%–18.1%; *P* = 0.05). Similarly, each 1 °C increase in minimum temperature was associated with a 4.9% increase in reported diarrheal incidence (95% CI: 2.8%–17.1%; *P* = 0.05). In contrast, rainfall, relative humidity, and seasonal categories were not significantly associated with reported diarrheal incidence in the Surkhet district (*P* > 0.05). In the Morang district, monsoon season was associated with a 21.3% higher reported diarrheal incidence relative to the non-monsoon reference period. Among the climatic variables included in the quasi-Poisson GLM, maximum temperature, rainfall, and relative humidity were not significantly associated with reported diarrheal incidence (*P* > 0.05). In contrast, minimum temperature showed a significant positive association, with each 1 °C increase associated with a 7% increase in reported diarrheal incidence (95% CI: 4.0%–17.3%; *P* < 0.05). In the Dhankuta district, monsoon season was associated with a 7.7% higher reported diarrheal incidence relative to the non-monsoon reference period (95% CI: 5.9%–32.3%; *P* < 0.05). Among climatic variables included in the quasi-Poisson GLM, maximum temperature, minimum temperature, and rainfall showed significant positive associations with reported diarrheal incidence. Specifically, each 1 °C increase in maximum temperature was associated with a 9.6% increase in reported diarrheal incidence (95% CI: 2.4%–38.7%; *P* < 0.001), while each 1 °C increase in minimum temperature was associated with a 10.3% increase in reported diarrheal incidence (95% CI: 3.3%–30.7%; *P* = 0.003). Rainfall also was positively associated with diarrheal incidence, with each 1 mm increase associated with a 0.9% increase in reported diarrheal incidence (95% CI: 0.02%–37.1%; *P* < 0.001). Relative humidity and other seasonal categories were not significantly associated with reported diarrheal incidence in the Dhankuta district (*P* > 0.05).

## 4 Discussion

This study is the first to assess the relationship between climate factors and childhood diarrheal disease and its seasonality across four districts of Nepal using long-term time-series analysis. The temporal trends observed in Fig. 2 suggest an apparent peak in diarrheal cases around 2010, followed by an overall decline through 2022, with particularly sharp reductions in Morang and Surkhet after 2015. The earlier peak may reflect a higher baseline diarrheal burden associated with lower sanitation coverage, greater reliance on unimproved water sources, and more limited WASH infrastructure in earlier years. The subsequent decline may plausibly reflect improvements in sanitation, access to safer drinking water, child health services, and broader public health intervention. However, an abrupt reduction in reported cases around 2015 also coincides with changes in Nepal's childhood illness reporting and recording systems. The DoHS reported implementation of Community-Based Integrated Management of Neonatal and Childhood Illness (CB-IMNCI) from fiscal year 2071/72 (Bikram Sambat calendar) (2014/15), accompanied by changes in the incorporation of health-facility and primary health-care/outreach-clinic data into HMIS and in the identification, classification and reporting of childhood diarrheal cases [21]. Although we found no evidence that the underlying definition of a new diarrheal case among children under five was changed, changes in reporting and recording practices may have contributed to the observed discontinuity. National HMIS data also showed a decline in reported diarrheal cases during this period [21]. Improvements in sanitation may have contributed to the longer-term decline, given the rapid expansion of the national open defecation free (ODF) campaign during this period. However, differences in the timing of sanitation achievements across districts make this an unlikely sole explanation for the abrupt reduction observed in both Morang and Surkhet. In addition, the 2015 Gorkha earthquake also does not provide a sufficient explanation for the observed pattern. Thus, the decline is likely multifactorial, involving both genuine changes in diarrheal burden and possible changes in routine reporting and ascertainment. Because facility-level information on reporting completeness and district-specific implementation of these changes was unavailable, the relative contribution of these factors and the reason for the greater magnitude of the decline in Morang and Surkhet cannot be determined. The post-2015 trends should therefore be interpreted with caution (Fig. 3).

**Fig. 3:**
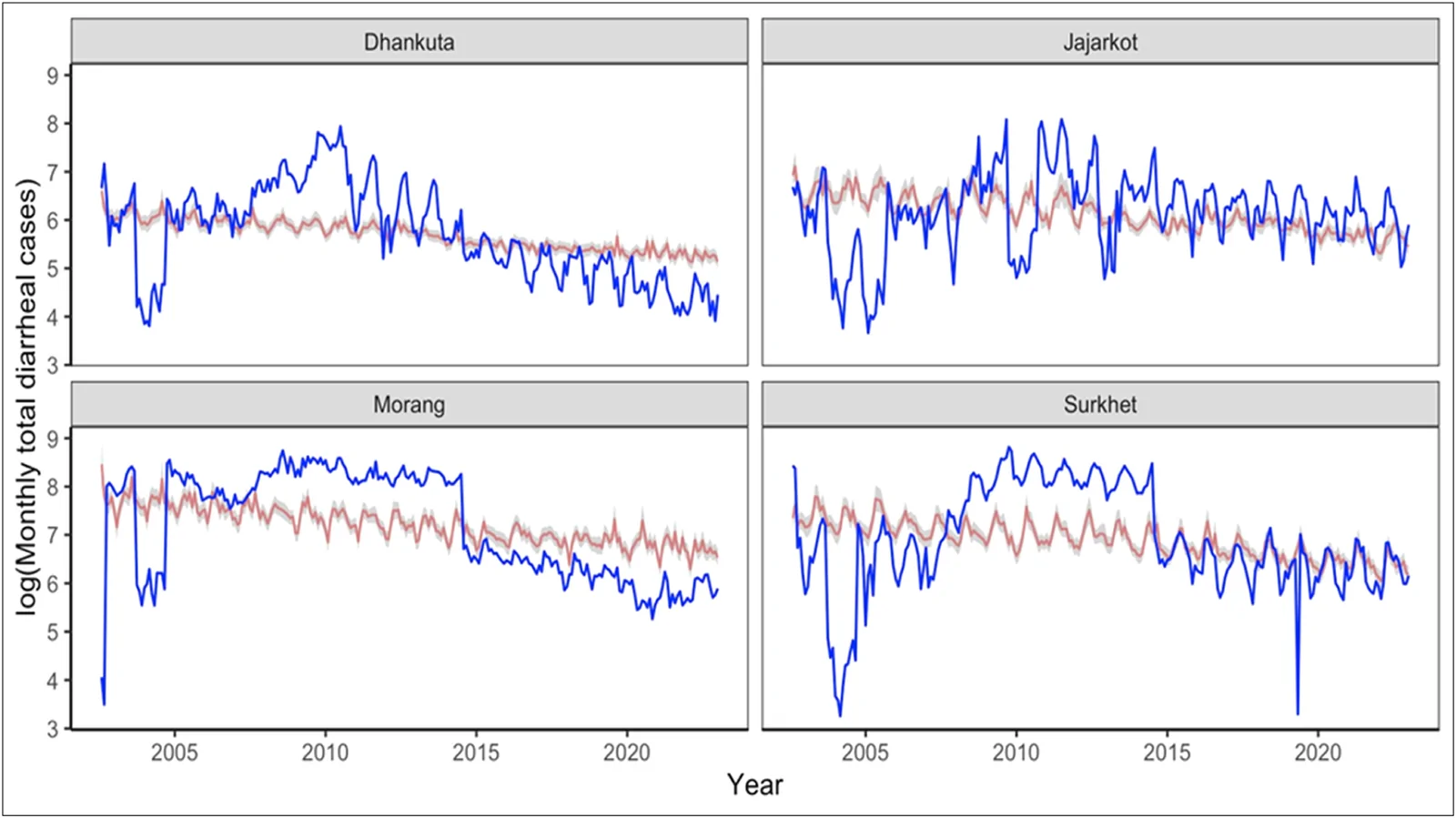
Time-series analysis of monthly total diarrheal cases (2002–2022). The figure displays time series plots of log-transformed monthly total diarrheal cases for four districts in Nepal: Dhankuta, Jajarkot, Morang, and Surkhet. Each plot compares the observed (raw) values of diarrheal cases (blue lines) with the predicted values from a generalized linear model (GLM) (red lines). The grey shading represents the standard errors of the predictions. The ***temporal trends*** of Dhankuta and Morang show a more noticeable decline in diarrheal cases over time, while Jajarkot and Surkhet exhibit more fluctuating trends without a clear decline.

The findings show that the incidence of diarrheal disease increases in all the districts during the monsoon season, which runs from June to September, compared to the pre- and post-monsoon seasons. Similar studies conducted in Bangladesh and the Philippines have found similar increased rates of diarrheal during the months with the highest monsoon rainfall [22,23]. The Jajarkot district showed a stronger monsoon-associated increase in diarrheal incidence compared with the other study districts. This finding may reflect the district’s limited access to safe drinking water, inadequate sanitation and hygiene infrastructure, and challenging hilly terrain, which can hinder water supply and sanitation development. Heavy monsoon rainfall and flooding may further compromise water quality and increase exposure to waterborne pathogens in vulnerable communities. Seasonal rainfall further contaminates water sources, while poor healthcare access and malnutrition increase susceptibility, particularly among children. Similar associations between poor WASH conditions, difficult terrain, and increased diarrheal disease risk during monsoon periods have been reported in other mountainous and resource-limited settings [17]. Additionally, the Jajarkot district historically has experienced significant diarrheal disease outbreaks, with numerous cases reported annually, highlighting the persistent public health burden in the region [24]. The observed associations between higher temperature, increased rainfall, and diarrheal incidence are consistent with findings from previous studies conducted in Nepal, China, Bangladesh, the Pacific Islands, and South America [12,14,18,[25], [26], [27]].These studies have reported a 3–15% increase in diarrheal cases per 1 °C rise in ambient temperature. Higher temperatures may enhance the survival and replication of enteric pathogens, while heavy rainfall can increase contamination of drinking water sources through runoff, flooding, and inadequate sanitation infrastructure. These mechanisms may contribute to increased transmission of diarrheal diseases, particularly in settings with limited water and sanitation services. Similarly, our findings suggest that higher maximum temperatures were associated with increased diarrheal incidence in some districts, with estimated increases ranging from approximately 5% to 10% per 1 °C rise in temperature. One possible explanation for this effect is that diarrheagenic pathogens, such as *Shigella* spp., have been shown to express virulence genes that trigger intestinal inflammation and toxin-induced fluid loss in response to temperature increases from 30 °C to 37 °C [10]. Temperature rise has the potential to enhance the survival rate and load of diarrhea-causing pathogens, particularly bacteria and protozoa, in their zoonotic host. This may result in prolonged pathogen transmission [9]. Likewise, warm weather increases the incidence of food poisoning-related diarrheal disease, as higher temperatures accelerate pathogen growth in food [28]. The temperature fluctuations may influence eating habits and personal hygiene, especially during hot days, a common occurrence in Nepalese communities. Consuming less hot, freshly prepared food may further facilitate the spread of bacteria and other infections [29]. Similarly, poor hygiene habits among parents and other caregivers during dry and hot weather may have contributed to higher-than-average infection transmission to children in our study.

The observed association between higher minimum temperature and increased diarrheal incidence is consistent with findings from previous studies. For example, a study conducted in Bangladesh reported that the monthly incidence of cholera increased by approximately 6% for every 1 °C rise in minimum temperature [30], supporting the patterns observed in our analysis. Warmer minimum temperatures may enhance the survival, growth, and transmission of enteric pathogens such as *Escherichia coli (E. coli)* and *Vibrio cholerae*, which are common causes of diarrheal disease. Elevated nighttime and minimum temperatures also may promote bacterial persistence in water and food sources, thereby increasing the likelihood of disease transmission.

In addition to the maximum and minimum temperatures, increased rainfall was associated with higher diarrheal incidence in the Dhankuta district, consistent with findings from studies conducted in Nepal and other regions worldwide [17,18,30,31]. Extensive examinations of nationwide datasets have revealed significant positive associations between heavy precipitation and waterborne illness outbreaks in the United States, Canada, and the United Kingdom, as well as between elevated rainfall and hospital admissions for diarrhea in Taiwan [[31], [32], [33], [34]]. In the Dhankuta district, heavy rainfall may cause water to run across the surface of grazing fields and seep into the drinking water supply, providing one probable explanation for the positive association between rainfall and the occurrence of diarrheal disease. Similarly, excessive rainfall can cause drainage to overflow into the sewage system, allowing large volumes of sewage-contaminated water to enter rivers [35]. In the Dhankuta district, where many communities rely on rivers and springs for their drinking water supply, rainfall-related contamination may increase exposure to enteric pathogens. These risks may be particularly pronounced in developing settings such as Nepal, where water supply and sanitation infrastructure remain limited in many areas.

Our results showed no positive association between the risk of diarrheal disease and relative humidity in any of the study districts. Our findings contradict some studies, which have shown that relative humidity contributed to an increased risk of diarrheal cases [33,[36], [37], [38]]. A study conducted in Singapore, a wealthier nation in Asia, found a 3% increase in diarrheal disease incidence for every 10% rise in relative humidity (IRR: 1.030, 95% CI: 1.004–1.057) [39]. Many other researchers have reported a negative association between rotavirus diarrhea in young children and relative humidity, which is consistent with our findings [[40], [41], [42]].

To summarize, while there is some variation across districts, we found a strong positive association between the frequency of diarrheal disease and the monsoon seasons, maximum and minimum temperatures and rainfall. Temperature had a significantly greater effect than other climatic variables in all the study districts. Focusing on four districts in Nepal's Koshi and Karnali regions, this study provides a more precise estimate of how climate factors influence the prevalence of diarrheal disease in the study areas, offering valuable insights into the impact of climate change. It is also important to consider temperature duration, as prolonged warm conditions may heighten pathogen growth and water contamination more than short-term fluctuations. This remains an important area for further investigation.

The study has several limitations. This study used routinely reported monthly diarrheal case counts from district surveillance systems, which may be affected by changes in reporting completeness, healthcare-seeking behavior, diagnostic practices, and surveillance quality over time. Although a generalized linear model with a quasi-Poisson distribution and log link function was used to account for overdispersion and reduce the influence of large differences in absolute counts, the analysis relied on absolute reported case numbers rather than normalized incidence rates or annual proportions. As a result, direct comparisons across years and districts should be interpreted cautiously, as observed temporal changes may reflect both true epidemiological variation and reporting-related factors. Future studies incorporating population-adjusted incidence rates, reporting completeness metrics, or proportional seasonal analyses may improve comparability across districts and time periods.

## 5 Conclusion

This study demonstrated a significant positive association between climatic factors and the incidence of diarrheal disease among children under five across nearly all districts. Notably, the impact of these factors varied markedly between districts, highlighting the need for district-specific interventions and public health strategies. To better plan, prevent, and adapt to climate-related waterborne diseases like diarrheal diseases across Nepal, local health sectors must understand the relationship between climatic factors and diarrheal disease incidence. Furthermore, the effects of climatic factors on the burden of diarrheal diseases should be addressed through public awareness campaigns and strengthening health surveillance systems to track climate-related disease patterns in all the districts of Nepal. Lastly, a well-integrated effort can help predict outbreaks, implement preventive measures, and reduce the overall burden of diarrheal diseases in the context of climate change.

## CRediT authorship contribution statement

**Bimala Dhimal:** Writing – review & editing, Writing – original draft, Visualization, Validation, Software, Resources, Project administration, Methodology, Investigation, Funding acquisition, Formal analysis, Data curation, Conceptualization. **Rijan Bhakta Kayastha:** Writing – review & editing, Visualization, Validation, Supervision, Resources, Project administration, Methodology, Formal analysis, Conceptualization. **Biraj Man Karmacharya:** Writing – review & editing, Visualization, Validation, Supervision, Resources, Project administration, Methodology, Formal analysis, Conceptualization.

## Declaration of competing interest

The authors declare that they have no known competing financial interests or personal relationships that could have appeared to influence the work reported in this paper.
